# 

**Published:** 1847-01

**Authors:** 


### CONTENTS

OF NO. I.

### ORIGINAL COMMUNICATIONS.

ESSAYS AND CASES.

Art. I.—Notes on Medical Matters in Paris. By David W. Yandell, M. D., of Louisville, Ky. - 1
Art. II.—A Case of Uterine Hydatids. By Francis H. Gordon, of Clinton College, Tenn. - - 9
Art. III.—Excision of the Inferior Maxillary Bone for Osteo-Sarcoma. By William H. Deaderick, M. D., of Athens, Tenn. - - - - 12
Art. IV.—A Case of Inversion of the Uterus. By E. Halley McCoy, M. D., of Harrisville, Ohio. - 16
Art. V.—A Case of Periodical Neuralgia. By James L. Taylor, of Trenton, Ohio. - - - 17
Art. VI.—A Case of Concussion of the Brain followed by Mental Derangement and Paralysis, successfully treated by Sulphuric Ether. By John Travis, M. D., of Melville, Tenn. - - - - 18

REVIEWS.

Art. VII.—Scrofula; its Nature, its Causes, its Prevalence, and the Principles of Treatment. By Benj. Phillips, F. R. S., Assistant Surgeon to the Westminster Hospital. Illustrated with an engraved plate. 8 vo. pp. 355. Lea & Blanchard. 1846. - - 20
Art. VIII.—The Water Cure for Hydropathy, from the British and Foreign Medical Review. By John Forbes, M. D., F. R. S., one of the Editors of the "Cyclopædia of Practical Medicine," Editor of the "British and Foreign Medical Review," etc., etc. Philadelphia: Lindsay and Blakiston. 1846. - - 38
ART. IX.—A Practical Treatise on the Diseases of Children. By JAMES COLEY, M. D., Member of the Royal College of Physicians in London, &c.; Author of a Treatise on the Remittent Fever of Infants, &c.; and of Essays on Phlegmonous Erysipelas, &c. 8 vo. pp. 414. Philadelphia: Ed. Barrington & Geo. D. Haswell, 1846. - - - - - - - 47

SELECTIONS FROM AMERICAN AND FOREIGN JOURNALS.

Iron in Cachexia - - - - - - - - 61
Lithotomy and Lithotrity. - - - - - - - 62
The Cæsarian Operation considered in a Theological Point of view. - - - - - - - - - - - 63
M. Velpeau on Flexions and Engorgement of the Uterus. - 65
Mesmerism in Surgery. - - - - - - - - 66
Insensibility during Surgical Operations produced by Inhalation. 67
Case of Concealed Delivery. - - - - - - - 74
Treatment of Varicose Veins. - - - - - - - 77
On the Ectrotic or Abortive Treatment of Gonorrhœa. - 78
Statistics. - - - - - - - - - - - 80
Two Cases of Double Vagina. - - - - - 81
Alleged Cure for Croup. - - - - - - - - 83
Combination of Carbonate of Iron with Sulphate of Quinine in Remittent Fever. - - - - - - - - 84
Medical Students in France and the United States. - - 84

ORIGINAL INTELLIGENCE.

Foreign Correspondence. - - - - - - 85
Xyloidine, or Gun-Cotton. - - - - - - - 68
Introductory Lectures. - - - - - - - - 90
Facts from Correspondents. - - - - - - - 92

